# RNA secondary structure prediction with convolutional neural networks

**DOI:** 10.1186/s12859-021-04540-7

**Published:** 2022-02-02

**Authors:** Mehdi Saman Booy, Alexander Ilin, Pekka Orponen

**Affiliations:** Department of Computer Science, Aalto University, Espoo, Finland

**Keywords:** RNA structure prediction, Deep learning, Pseudoknotted structures

## Abstract

**Background:**

Predicting the secondary, i.e. base-pairing structure of a folded RNA strand is an important problem in synthetic and computational biology. First-principle algorithmic approaches to this task are challenging because existing models of the folding process are inaccurate, and even if a perfect model existed, finding an optimal solution would be in general NP-complete.

**Results:**

In this paper, we propose a simple, yet effective data-driven approach. We represent RNA sequences in the form of three-dimensional tensors in which we encode possible relations between all pairs of bases in a given sequence. We then use a convolutional neural network to predict a two-dimensional map which represents the correct pairings between the bases. Our model achieves significant accuracy improvements over existing methods on two standard datasets, RNAStrAlign and ArchiveII, for 10 RNA families, where our experiments show excellent performance of the model across a wide range of sequence lengths. Since our matrix representation and post-processing approaches do not require the structures to be pseudoknot-free, we get similar good performance also for pseudoknotted structures.

**Conclusion:**

We show how to use an artificial neural network design to predict the structure for a given RNA sequence with high accuracy only by learning from samples whose native structures have been experimentally characterized, independent of any energy model.

**Supplementary Information:**

The online version contains supplementary material available at 10.1186/s12859-021-04540-7.

## Background

### The RNA structure prediction problem

RNA is a highly versatile molecule of life: it has several key roles in the essential cellular processes of gene expression and regulation, carries cellular signals, and serves as a multi-purpose catalyst. It is a linear polymeric molecule constituted of elementary nucleotide units with bases adenine (A), cytosine (C), guanine (G) and uracil (U), bound to a sugar-phosphate backbone. RNA molecules, which are natively single-stranded, fold upon themselves to create biologically active 3D conformations, following mostly (but not completely) similar Watson-Crick base pairing rules as DNA: adenine pairs with uracil (A-U) and guanine with cytosine (G-C), but often also with uracil (the G-U *wobble pair*). To understand, and eventually control, this critical function-forming process, it is important to be able to predict how a given nucleotide sequence (the *primary structure*) folds upon itself to create a base-pairing *secondary structure* and eventually the geometric 3D *tertiary structure*. Because predicting the final tertiary structure is extraordinarily difficult, much research has focused on trying to resolve the intermediate problem of secondary structure formation.

In simple cases, RNA secondary structures exhibit a clean hierarchical arrangement composed of blocks of matching base-pairs (*stem segments*) interspersed with intervals of unpaired bases (*loops*), analogous to a well-parenthesised string in a formal language. In fact, one standard representation for these basic structures is the *dot-bracket notation*, where the bases are enumerated from the 5’-sugar end of the backbone towards the 3’-sugar end: each base initiating a pair is denoted by an opening parenthesis, the matching closing base by a closing parenthesis, and the unpaired bases by dots. The situation is, however, significantly complicated by base-pairs that break this hierarchical arrangement, so called *pseudoknot* connections. Theoretically, an optimal non-pseudoknotted secondary structure for a given sequence can be found efficiently by a dynamic programming approach, whereas the problem becomes NP-complete when pseudoknots are allowed [1].

### Related work

Most secondary structure prediction approaches propose some scoring function and strive to find appropriate structures with respect to this function. In the common case where the score is based on an energy model, the goal is either to determine a minimum free energy (MFE) structure in the given model, or sample structures according to a corresponding Boltzmann probability distribution.

#### Energy-based algorithmic methods

These methods find a thermodynamically minimum free energy structure for a given sequence and an energy model. Zuker [2, 3] proposed a basic dynamic programming approach to find an MFE structure by aggregating locally optimal structural elements with respect to a proposed energy model. Later on, Turner [4, 5] presented a more comprehensive “nearest neighbour” energy model, which became the core for many other methods originating from the Zuker algorithm, such as UNAFold [6], RNAStructure [7] and Vienna RNAfold [8], the latter tuning the energy parameters somewhat. Lyngsø and Pedersen [1] showed that finding MFE structures in a given energy model becomes NP-complete when pseudoknots are allowed. Hence, algorithmic methods based on dynamic programming cannot cover pseudoknots without compromising their efficiency. Some methods such as IPknot [9] and ProbKnot [10] use heuristics to predict also pseudoknotted structures.

#### Energy-based learning methods

The MFE structure for a sequence, given an energy model, is not necessarily the desired target structure. Energy models are not perfect because the thermodynamic parameters are calculated experimentally from many yet not sufficient number of samples. The ContraFold method [11] tries to learn new parameter sets and find the structure with respect to them, although the optimization is still with a dynamic programming algorithm.

#### Deep learning methods

CDPFold [12] uses a convolutional neural network to predict a scoring matrix that is then fed to a dynamic programming algorithm to extract the dot-bracket structure. It can only predict non-pseudoknotted structures due to being limited to the dot-bracket notation, and also is not time-efficient for sequences longer than a few hundred bases because of the dynamic programming post-processing. Recently, E2Efold [13] proposed a deep neural network that outputs scores for all possible pairings in an RNA sequence and a differentiable post-processing network that converts the scores into a secondary structure. The score network of E2Efold had an architecture based on transformers [14] and convolutional layers. The post-processing tool was designed by convex relaxation of a discrete optimization problem to a continuous one. SPOT-RNA [15] is a deep learning model based on convolutional layers and custom 2D-BLSTM layers. This model considers also triplets (bases connected to two others) and non-canonical pairings. However it is limited to sequences shorter than 500 nucleotides (nt) due to the complexity of the model and memory limit. MXFold2 [16] is the most recent model which contains one-dimensional and two-dimensional convolutions and recurrent BiLSTM layers. The output has four different scores for each pair including helix stacking, helix opening, helix closing and unpaired region. The model that we propose in this paper is conceptually much simpler than the previous models, yet it results in very competitive performance.

#### Related problems

Learning-based methods dominate in related structure prediction problems. For example EternaBrain [17] uses CNN and [18] uses reinforcement learning to address the RNA sequence design (inverse folding) problem. As another example, the recently proposed AlphaFold [19] set the new state of the art in predicting structures for proteins. The algorithm contains multiple deep learning components such as variational autoencoders, attention mechanism and convolutional networks.

### Problem definition

**Fig. 1:**
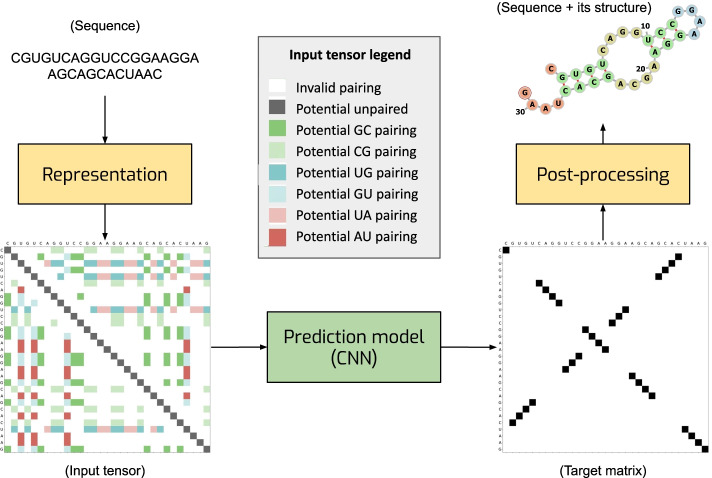
General illustration of our solution. We represent an RNA sequence as two-dimensional map with 8 channels with one-hot encoding (each color in the input tensor represents a one-hot vector of size 8. The gray box shows what each color means. for example, dark green is a potential GC pairing which is [0,0,0,0,0,0,1,0]). We process the map with a convolutional network which produces a score matrix for all possible pairings. Finally, we convert the score matrix into the RNA secondary structure

The problem of predicting the secondary structure of an RNA can be formulated as follows. Given a sequence of bases $q=(q_1, q_2, \ldots , q_L)$, where each base $q_i$ can take one of the four values A, U, C, G, the task is to predict a set of pairings $\{(q_i, q_j)\}$ that define the secondary structure. For example, given a sequence CGUGUCAGGUCCGGAAGGAAGCAGCACUAAC, one needs to predict the pairings $(q_2, q_{27}), (q_3, q_{26}), (q_4, q_{25}), (q_5, q_{24}), (q_{10}, q_{19}), (q_{11}, q_{18}), (q_{12}, q_{17})$ which define the structure shown in Fig. 1.

There are a set of constraints that need to be satisfied:

- There are six possible types of pairings: (A, U), (U, A), (U, G), (G, U), (G, C), (C, G) (Watson-Crick and wobble pairing types).
- Each base can be either pairs with a single other base or unpaired. If base *i* is paired with base *j*, base *j* is paired with base *i*.
- The minimum distance for pairing is 3, that is $|i-j| \ge 3$.

## Materials and methods

### Representing RNA sequences and secondary structure targets as tensors

The key component of our approach is the way we encode RNA sequences. We represent an RNA sequence *q* of length *L* as an $L \times L \times 8$ tensor *X* which can be viewed as a two-dimensional $L \times L$ map with eight channels (see the input tensor Fig. 1). An eight-dimensional vector of features in location (*i*, *j*) of *X* is a one-hot representation of eight possible relations between bases $q_i$ and $q_j$ in positions *i* and *j*:

- Six channels indicate that base $q_i$ can pair with base $q_j$, that is pair ($q_i, q_j$) is one of the six possible combinations of bases (A, U), (U, A), (U, G), (G, U), (G, C), (C, G).
- One channel is used to indicate that $i=j$, i.e. this channel is set to ones only for the positions on the main diagonal of map *X*. The purpose of this channel is to ease detecting unpaired bases which we encode with non-zero elements on the diagonal of the target matrix.
- One channel indicates that a pairing between bases *i* and *j* is not possible due to a non-valid combination of bases, too short distance between two bases, or any other constraint.

We formulate the target for the model output as a binary $L \times L$ matrix *T* in which the *ij*-th element $t_{ij}=1$ if bases *i* and *j* are paired and $t_{ij}=0$ otherwise. $t_{ii}=1$ means that base *i* is unpaired (see the target matrix Fig. 1).

The advantage of the proposed representation is that it makes it equally easy to predict local and long-distance pairings. Local pairings are represented by non-zero elements in maps *X* and *Y* that are close to the main diagonal (see the target matrix in Fig. 1). Long-distance pairings correspond to locations in *X* and *Y* that are farther away from the main diagonal. Both types of structures can be easily detected by processing the input *X* with a convolutional neural network (CNN). CNN is also a powerful tool for detecting stem segments: blocks of consecutive bases paired with another block of bases. In our matrix representation, such pairings are represented by a sequence of non-zero elements in matrix *Y* which are either parallel or orthogonal to the main diagonal. These patterns can be easily detected with a CNN. Due to weight sharing, CNNs can process sequences of varying length and the processing is equivariant to translations of the input sequence. These are useful properties for our application.

### Prediction model

We represent each sequence as a 3-dimensional tensor (input tensor in Fig. 1) which is the input of our prediction model. The output of the network is a 2-dimensional matrix (Target matrix in Fig. 1) in which each element at (*i*, *j*) position shows the score for having (*i*, *j*) pairing in the predicted structure. Then, we extract the structure using our post-processing method.

**Fig. 2:**
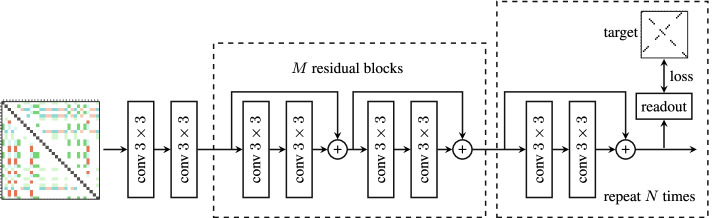
The architecture of the prediction model. “conv” denotes a convolutional layer followed by batch normalization and LeakyRelu nonlinearity. The readout layer is another conv block followed by a convolution layer both with kernel size 1. The loss is computed after each residual block with skip connections. The left dashed block, a *residual block*, is repeated *N* times with different weights, whereas the right one, a *shared residual block*, is used *M* times with the same weights

The prediction model takes an $L\times L \times 8$ tensor *X* as input and produces an output *Y* of shape $L\times L$. The model starts with two convolution blocks followed by *M* residual blocks with skip connections and *N* residual blocks with shared weights (see Fig. 2). Each conv $k\times k$ block is a convolutional layer with a $k\times k$ kernel followed by batch-normalization and LeakyRelu activation function. To keep the size after each convolutional block unchanged, we have applied the required padding. We repeat the residual block (left dashed block) *M* times in series. Output of block 1 will be the input of block 2 and finally output of block *M* goes to the first shared residual block (right dashed block). We have *M* residual block (with different weights) and only one shared residual block which we repeat *N* times (with the same weights) like a recurrent model. Output of the shared residual block would be the input of itself in the next iteration. Readout has a conv$1\times 1$ block and then a single convolution layer with kernel size 1. All the conv blocks have 32 output channels except the last conv$1\times 1$ in the readout block which has 1.

We encourage the network to arrive at the correct solution as fast as possible by computing the loss after each shared residual block. The model output $Y_n$ after the *n*-th shared residual block is computed using a readout module which is a convolutional block followed by a convolution layer with one output channel. The loss function penalizes the difference between $Y_n$ and the target matrix *T*. We use the mean-squared error as the loss:

$$\begin{aligned} l_n = \frac{1}{|V|} \sum _{ij \in V} (y_{ij}^{(n)} - t_{ij})^2 \end{aligned}$$

where $y_{ij}^{(n)}$ is the *ij*-th element of $Y_n$ and *V* is a set of all pairs except for pairs with a non-valid combination of bases, too short distance between the bases or any other constraints. The mean-squared error was chosen because it produced the best results among other alternatives that we tried. The final loss is the average of the *N* intermediate loss values $l = \frac{1}{N} \sum _{n=1}^N l_n$.

### Post-processing

The output of the model is an $L \times L$ matrix which needs to be converted into a set of pairings that represent the secondary structure of an RNA sequence. We have used two alternative approaches for post-processing. Both of the approaches return a single structure extracted from the output score matrix. Also sub-optimal structures can be extracted with a different post-processing approach, although we have not tested it as our focus in this paper is on single structure prediction. Another possibility for post-processing would be dynamic-programming based methods (essentially Zuker’s algorithm using output score values in place of energy values) but these get very complicated if one wishes to consider pseudoknots.

In the first approach that we call *Blossom post-processing,* we extract a secondary structure in which each base is either paired to a single other base or unpaired (this condition holds for all RNA structures in the training datasets that we considered) by the technique of *maximum weighted matching in graphs*. The model output $Y=Y_N$ is interpreted as a weighted adjacency matrix of a weighted graph in which the nodes correspond to bases and the weights of the edges reflect the chance that there is a pairing between the corresponding bases according to the model.^1^ Our goal is to find a set of edges without common nodes and with the option of including self-loops (an unpaired base is considered to be paired with itself) such that the sum of the weights is maximised. We solve this problem using the classical *Blossom algorithm* [20].

We used available implementations of the Blossom algorithm that do not support self-loops. To overcome this limitation, we created a graph which contains two copies of the original weighted graph with the self-loops excluded and with additional connections between each pair of nodes that represent the same node in the original graph. The weights of the additional connections are the weights of the corresponding self-loops, multiplied by two. The node-matching edges eventually chosen by the algorithm are taken to correspond to the pairings in the structure. To make the modeling more clear, there is a simple example in Additional file 1.

This post-processing algorithm guarantees that each base is either paired with a single other base or unpaired. A challenge with this method is, however, that it is computationally quite expensive, especially for long sequences. It has time complexity $O(|E||V|^2)$, where $|V|=L$ is the number of nodes in the graph and |*E*| is the number of edges. If one considers only *k* largest-weight edges for each node, the complexity is reduced to $O(kL \times L^2)=O(kL^3)$. We tried the post-processing with different values for $k=1,\ldots ,L$, and observed that values of $k > 3$ no longer improve the output quality, despite the increased runtime.

In the second approach, we connect base *i* to base *j* if the corresponding value $y_{ij}$ of the model output is the largest one in the *i*-th row of *Y*. This algorithm runs in time $O(L^2)$ but it often produces invalid structures, because it does not guarantee symmetric pairings. The method, nevertheless, yields similar results in terms of precision and recall as Blossom post-processing (see Table 2), and so we use it when we tune the hyperparameters of the model. We call this algorithm *Argmax post-processing*. The default post-processing method is Argmax unless we indicate that Blossom has been used.

## Results

Learning-based RNA structure prediction methods can be evaluated in two different aspects, sequence-wise cross-validation and family-wise cross-validation. In the former, the test set has same families as the training set but the sequences are not redundant; however structural similarities between train and test may occur. In the latter, the test set comes with samples from new/different families than the training set; structural similarities may still occur but are less likely. Our focus in this paper is on sequence-wise cross-validation.

### Datasets

There are three commonly used datasets for RNA structure prediction.

**Fig. 3:**
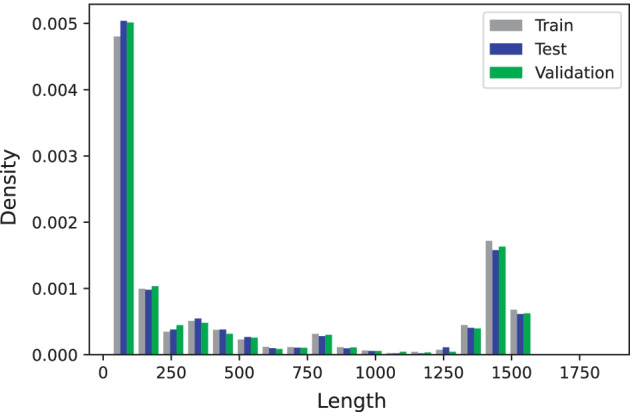
RNAStrAlign dataset lengths density for train, test, and validation sets. We use density instead of absolute number of samples because of different set sizes

1. RNAStralign [21] contains 37149 structures from eight RNA families (16S, 5S, Group I Intron, RNaseP, SRP, telomerase, tmRNA, tRNA) with sequence lengths varying between 30 and 1851 nucleotides (nt). For sequences with multiple secondary structures, we randomly kept only one target secondary structure and therefore retained only 30451 samples. There are no redundant sequences but there are redundant structures. As there are many sequences in one particular RNA family, structural similarities exist in this dataset. We split the dataset into 80% training (RSA-tr), 10% validation (RSA-vl), and 10% test (RSA-ts) sets (exactly as suggested in [13]) so that each RNA family had approximately the same representative fraction in each set as in the full dataset. Using the exact same splits from [13] not only leads to not having any redundancies for the sequences but also makes the comparison fair. Figure 3 shows the frequency of different lengths in this dataset in which the proportions are the same for all train, test, and validation sets. We use this dataset for sequence-wise cross-validation evaluation.
2. ArchiveII [22] contains 2975 samples with sequence lengths between 28 and 2968 nt from 10 RNA families (two additions to the RNAStrAlign families, 23S and Group II Intron). We only tested our trained model RSA-tr with this dataset without any fine-tuning to evaluate it on a completely different sample set and compare with other methods. Same as before, we use the exact same test set suggested in [13] that has no redundancy for the sequences.
3. bpRNA [15] contains 12119 samples shorter than 500 nt from several RNA families created from rfam 12.2. We compare our model with SPOT-RNA and MXFold2 with training on TR0 (with 10814 samples) and testing on TS0 (with 1305 samples) as the exact same split as [15] in which the similarity CD-HIT-EST>0.8 are removed. Another version of this dataset, bpRNA-new, is introduced in [16] which has 1500 RNA families from rfam 14.2 which are not in the bpRNA. This is the only dataset that we use for family-wise cross validation (training on TR0 and testing on bpRNA-new).

We evaluated the trained models using average precision, recall and F1-score, where precision reflects “how correct are our predicted pairings”, recall shows “how many of the target pairings our model could predict”, and F1-score is a harmonic average of the first two.

We trained the following variants of the proposed model.

1. CNNFold has $M=2$ residual blocks and $N=2$ shared residual blocks trained for 30 epochs on the whole trainset (RSA-tr).
2. CNNFold-600 has $M=2$ residual blocks and $N=2$ shared residual blocks but trained for 400 epochs on the samples shorter than 600 nt from RSA-tr.
3. CNNFold-600-big has $M=10$ residual blocks and $N=2$ shared residual blocks trained for 45 epochs on samples shorter than 600 nt in RSA-tr. Due to the memory limit, we cannot use this model for long sequences.

We trained three different models using the Adam optimizer with learning rate 0.005. To avoid problems caused by the limited size of the GPU memory, we used mini-batches with varying sizes. We used only one sequence in a mini-batch for sequences longer than 1000 nt, while we used up to 16 samples in mini-batches containing shorter sequences. CNNFold and CNNFold-600-big have 95k and 317k parameters respectively while E2Efold and SPOT-RNA have 719k (almost 2 times more) and 1746k (almost 4 times more) parameters respectively.

While tuning the model, we found that CNNFold works slightly worse on short sequences ($L \le 600$) than CNNFold-600. CNNFold-600-big outperforms the other two models on short sequences. These results are presented in Table 1. Eventually, we use CNNFold-600-big to process sequences with $L \le 600$ and CNNFold to process sequences with $L > 600$. We call this ensemble of the two models CNNFold-mix.

**Table 1:** Results for our model trained on different parts of the RNAStrAlign training set (trained on RSA-tr and tested on RSA-vl)

	F1, all data	F1, L ≤ 600	Weighted F1, all data
CNNFold-600-big	–	**0.970**	–
CNNFold-600	0.900	0.948	0.812
CNNFold	**0.916**	0.928	0.842
CNNFold-mix	0.936	0.970	0.863

Bold values are the best result in each column

**Fig. 4:**
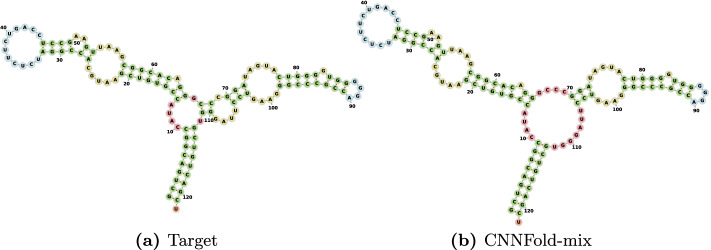
Visualization for E00001 from 5sRNA family. **a** Is the target secondary structure and **b** is our prediction produced by CNNFold-mix with 93.4% accuracy. Structure diagrams are generated by the Forna [23] tool

The results on the RNAStrAlign dataset (train and test on this dataset) indicate that our model achieves significant improvements compared to the present state of the art on this dataset (see Table 2). For example, the fraction of undetected pairings is only 0.093 for our model, which is less than two-fifths of the value 0.127 achieved by MXFold2. Our model achieves an impressive F1-score of 0.936 which is substantially higher than 0.868 of the previously best method on RNAStrAlign dataset. We cannot compare the results with SPOT-RNA on this dataset since the training module is not provided. We will do the comparison with this method on other datasets. Figure 4 shows one randomly picked sample from 5S family. There are two other examples in the supplementary materials (see Additional files 2 and 3). Not only all the predictions are visually close to their target structures, but they are also valid structures due to our Blossom post-processing method.

**Table 2:** Results on the RNAStrAlign dataset (sequence-wise CV)

	Precision	Recall	F1	Prec (S)	Recall (S)	F1 (S)	Weighted F1
Mfold [6]	0.450	0.398	0.420	0.463	0.409	0.433	0.366
RNAfold [8]	0.516	0.568	0.540	0.533	0.587	0.558	0.444
RNAstructure [7]	0.537	0.568	0.550	0.559	0.592	0.573	0.471
LinearFold [24]	0.620	0.606	0.609	0.635	0.622	0.624	0.509
CDPfold [12]	0.633	0.597	0.614	0.720	0.677	0.697	0.691
CONTRAfold [11]	0.608	0.663	0.633	0.624	0.681	0.650	0.542
E2Efold [13]	0.866	0.788	0.821	0.880	0.798	0.833	0.720
MXfold2 [16]	0.864	0.873	0.868	0.876	0.884	0.879	0.694
CNNFold + Argmax	0.955	0.861	0.900	0.955	0.872	0.902	0.812
CNNFold-mix + Argmax	0.956	0.912	0.932	0.958	0.915	0.934	0.863
**CNNFold-mix + Blossom**	**0.975**	**0.907**	**0.936**	**0.978**	**0.909**	**0.938**	**0.872**

Bold values are the best result in each column

“(S)” indicates the results when one-position shifts are allowed, that is for a base pair (*i*, *j*), the following predictions are also considered correct: $(i+1,j)$, $(i-1,j)$, $(i,j+1)$, $(i,j-1)$. The numbers for the comparison methods are from [13]. All trainable models have been trained on RSA-tr

**Fig. 5:**
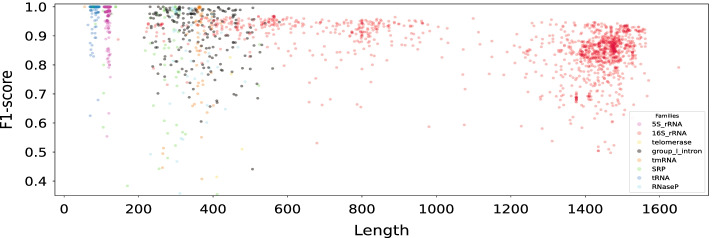
Scatter plot of the per-sequence F1-scores against the sequence lengths. Each point represents a sample and the model is CNNFold-mix. Colours indicate sequences from eight RNA families from RNAStrAlign

Our model performs very well on both short and long sequences. One indication of this are the results presented in Table 1. To emphasize the performance on longer sequences, similarly to [13], we computed a weighted average of the F1-scores where the weight for a sequence of length $L_k$ is $w_k = L_k /\sum _k L_k$. The weighted F1-score (see the last column of Table 2) indicates that our model works much better on long sequences compared to the previous methods. Even MXfold2 as the best competitor on this dataset, does not predict long sequences that well (with 0.694 as the weighted f1 score). Figure 5 shows a more in-detail scatter plot in which each point represents a sample with its length and F1-score.

CNNFold-mix outpeforms other methods in predicting pseudoknotted structures on RNAStrAlign dataset. Out of the 3707 samples in RSA-ts, 1413 are pseudoknotted, and we achieved an F1-score 0.857 on this subset compared to 0.71 for E2Efold and 0.472 for RNAstructure. Although pseudoknotted structures are presumed to be more complex, CNNFold predicts them almost as well as non-pseudoknotted ones. A scatter plot of the pseudoknotted samples with respect to their lengths and their F1-score is presented in fig Additional file 4. CNNFold-mix predicts 1412 out of 1413 pseudoknotted structures as pseudoknotted, compared to 1312 for E2Efold.

As another popular benchmark, we evaluated our model performance on the popular ArchiveII dataset [22] (summarised in Table 3). Since MXfold2 does not consider pseudoknots, it is trained on a subset of RSA-tr with excluding pseudoknots and samples longer than 600 nt. To have a fair comparison, we retrained our model, CNNFold600-big, on the same subset.

**Table 3:** Performance on the ArchiveII dataset (sequence-wise CV)

	Precision	Recall	F1
Mfold	0.428	0.383	0.401
CDPfold	0.557	0.535	0.545
RNAstructure	0.563	0.615	0.585
RNAfold	0.565	0.627	0.592
LinearFold	0.641	0.617	0.621
CONTRAfold	0.607	0.679	0.638
E2Efold	0.734	0.660	0.686
MXFold2 [16]	0.790	0.815	0.800
**CNNFold-mix**	**0.928**	**0.879**	**0.897**

Bold values are the best result in each column

All trainable models have been trained on a subset of RSA-tr with removing pseudoknotted structures and samples longer than 600 nt

The SPOT-RNA group [15] did not report results on the ArchiveII dataset and training their model is not straightforward due to lack of access to their training module and the 500 nt length limit. We trained our model on TR0 and compare it with SPOT-RNA and MXFold2 on TS0. Table 4 shows the comparison on the bpRNA dataset. All three methods have close F1-scores while CNNFold and SPOT-RNA have better precision and MXFold2 has higher recall. Having 1500 different RNA families make this dataset harder for our model, since it it tries to capture the patterns in families and use them in the prediction.

**Table 4:** Performance on the bpRNA dataset (sequence-wise CV)

	Precision	Recall	F1
SPOT-RNA	**0.652**	0.578	**0.597**
MXFold2	0.520	**0.682**	0.575
CNNFold-mix	0.640	0.566	0.582

Bold values are the best result in each column

All models have been trained on TR0 and tested on TS0. Comparison numbers are from [16] as we use the exact same splits for train and test

Our performance highly depends on the specific RNA families (with Telomerase and SRP being the hardest families for our model) since our model is just learning the prediction from samples in an end-to-end fashion without using the notion of energy or any other external features. In Fig. 5, we use different colours to show F1-scores for sequences from eight RNA families from the RNAStrAlign test set. Number of samples in each family and their lengths, in addition to average F1-scores for different RNA families are shown in Table 5 for the RNAStrAlign and ArchiveII datasets.

**Table 5:** F1-scores obtained with CNNFold-mix for different RNA families

Family	RNAStrAlign			ArchiveII		
	Lenghts	#Samples	F1-score	Lenghts	#Samples	F1-score
All	30–1851	30451	0.932	28–2968	3975	0.897
16SrRNA	54–1851	11620	0.855	73–1995	110	0.639
5SrRNA	104–132	9385	0.992	102–135	1283	0.972
tRNA	59–95	6443	0.996	54–93	557	0.937
Grp 1 Intron	163–615	1502	0.903	210–736	98	0.722
SRP	30–553	468	0.787	28–533	928	0.798
tmRNA	102–437	572	0.830	102–437	462	0.871
RNaseP	189–486	434	0.832	120–486	454	0.824
telomerase	382–559	37	0.615	382–559	37	0.755
23SrRNA	–	–	–	242–2968	35	0.489
Grp 2 Intron	–	–	–	619–780	11	0.591

Model has been trained on RSA-tr and tested on RSA-ts and ArchiveII

Although the focus of this work is sequence-wise cross validation, we have compared our method with others with training on TR0 and testing on bpRNA-new. Our method, MXFold2, SPOT-RNA, E2Efold, and RNAfold have 0.496, 0.632, 0.592, 0.036, and 0.617 F1-scores respectively. E2Efold completely failed to predict on this dataset while MXFold2 shows the best performance in family-wise CV.

For sequence-wise cross validation, CNNFold performs better than the other methods on RNAStrAlign and ArchiveII datasets and similar to SPOT-RNA on bpRNA. It has less parameters than E2Efold, MXfold2 and SPOT-RNA which let us train it on longer sequences as well. That is why it has a good performance on long sequences as well (0.872 for weighted f1 score compared to 0.72 for E2Efold on RNAStrAlign). CNNFold predicts psuedoknotted structues in RNAStrAlign better than E2Efold while MXfold2 does not support psuedoknots.

CNNFold has some difficulties for family-wise cross validation (on bpRNA-new) since it is agnostic about the folding problem and tries to learn the sequence-structure mapping only from the samples (and not any external features line free energy). It cannot generalise the prediction from samples in RNAStrAlign to samples from a new RNA family. MXFold2 integrates free energy values in the model which helps for the generalization. All in all, CNNFold predicts decently if the sequence belongs to a family in trainset, otherwise, the prediction might be problematic.

### Time analysis

**Fig. 6:**
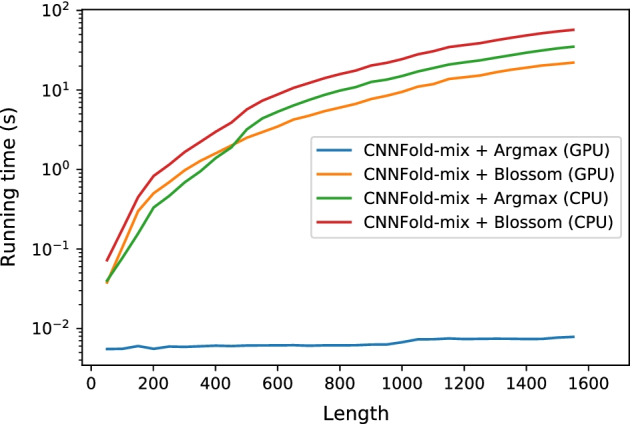
Running time on CPU and GPU for different lengths. Samples are from RSA-ts. Note that we run Blossom on CPU in all cases

It is important to analyze how our model scales with respect to sequence length. Figure 6 shows the running time on CPU (Intel Xeon Gold 6230, 2.10Ghz) and GPU (NVIDIA Tesla V100 SXM2 with 32GB RAM) for CNNFold-mix with both argmax and Blossom post-processing. Since we could not find any GPU implementation for the Blossom algorithm, in the “CNNFold-mix + Blossom (GPU)” Blossom has been run on CPU. The CNN model on the GPU is quite fast as it does not have huge number of parameters and runs convolutions in parallel. The Blossom algorithm has cubic time complexity and thus gets sluggish for long sequences (around 20 s for a sequence with 1400 nt).

### Model parameters

In CNNFold and CNNFold-600, $M=N=2$ but in CNNFold-600-big $M=10$ and $N=2$. Th learning rate for all of our training is 0.005. The loss function is MSE between the predicted and target matrices as we represent all structures in a 2-dimensional form and the loss will be calculated after each shared residual block. In Blossom we keep $k=3$ maximum scores for each base (each row in the score matrix). Maximum number of samples in a batch is 16 but it can be less for longer samples.

For other methods we used Mfold v2.3, CDPfold published on May 2019, RNAStructure v6.2, Vienna RNAfold v2.4.13, LinearFold published on July 2019, CONTRAfold v2.02, E2Efold published Feb 2020, MXFold2 published on Feb 2021 and SPOT-RNA published on Nov 2019. For all trainable methods in comparisons, we have mentioned the training set.

## Discussion

We have proposed a new learning-based method CNNFold for RNA secondary structure prediction independent of any energy model. Our results show that CNNFold significantly outperforms state-of-the-art methods in sequence-wise cross-validation comparisons, MXfold2 on RNAStrAlign and ArchiveII datasets while achieving comparable results as the SPOT-RNA on the bpRNA. Although the CNNFold model is less complex than the others (without any LSTM-like layer and with fewer parameters), it shows an outstanding performance thanks to the representation in which possible pairings are considered instead of only the sequence. It works also on pseudoknotted structures without any changes, and predicts them quite well.

CNNFold cannot generalise to samples from completely new families (compared to its trainset) because it tries to learn the prediction only using samples without any thermodynamic consideration like free energy. Adding some expert knowledge (like the free energy integration in MXfold2) to our agnostic model would help for the generalisation.

We believe that the method can be improved further to achieve an even better accuracy. One possibility is to take into account the length of the RNA sequence. This may have a positive impact on accuracy as the ensemble of models trained on different sequence lengths achieved the best performance in our experiments. Considering the RNA family might help as well, since our model performance depends on families. One can consider the family as an input/given, or train the model in such a way to detect the family first and then the structure given the predicted family.

Another way to extend this work is trying to generate sub-optimal structures as we have the score matrix from our network. With a different post-processing method we can generate more than one structure. As the model predicts structures that are not MFE, it might be interesting to check the free energy difference between the prediction and the target.

An important line of future research is to understand the limitations of the proposed method and other learning-based algorithms for RNA secondary structure prediction. The accuracy of the model is superb but it is important to understand how well the model can predict structural elements which are biologically important. For example, pseudoknots are difficult to predict, but missing any of them may have a significant effect on the functional properties of an RNA structure.

The ultimate goal of this line of research is to design new RNA sequences with the required functional properties. The results presented in this paper suggest that the proposed model can be a useful building block towards achieving this goal. It remains to be seen how well the model generalises to completely new RNA sequences that can be proposed in the design process. It may also be useful to extend the model to support multiple secondary structure predictions for a given sequence. This way one can increase the chance of finding RNA structures with the required functional properties.

## Supplementary Information

**Additional file 1**. An example for how we model the problem for Blossom.Assume the simple structure in the gray box (top left). The graphrepresentation for the pairings is shown in the left graph as eachpairing is an edge between two vertices. As the Blossom cannothandle self-loops (which are the unpaired bases), we create a copyof the graph (shown in the right graph with gray nodes) and converteach self-loop into an edge between the node and its copy. For thesake of simplicity, we set *k* = 1 in this example.

**Additional file 2**. DQ923214 from 16sRNA family, accuracy of CNNFold-mix is 97.1\% F1-score.(a) is the target structure and (b) is our prediction.

**Additional file 3**. CP000076 with pseudoknots, F1-score of CNNFold-mix is 90.8\% F1-score.(a) is the target structure and (b) is our prediction.

**Additional file 4**. Scatter plot of the per-sequence F1-scores against the sequence lengths for pseudoknotted structures. Each point represents a sample and the model is CNNFold-mix. Coloursindicate sequences from 6 RNA families from RSA-ts.

## Data Availability

All codes, datasets, and trained models is available on the Github via following link: https://github.com/mehdi1902/RNA-secondary-structure-prediction-using-CNN.

## Abbreviations

CNN: Convolutional neral network
nt: Nucleotide
MFE: Minimum free energy

## Footnotes

1 To reduce the computational cost, we retain only $k=3$ edges of maximum weight for each node.
