# Supplementary figures and images for: RNA secondary structure prediction with convolutional neural networks
Source: BMC Bioinformatics. 2022 Feb 2;23:58. doi: 10.1186/s12859-021-04540-7 (PMC8812003; doi:10.1186/s12859-021-04540-7)

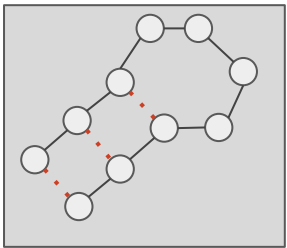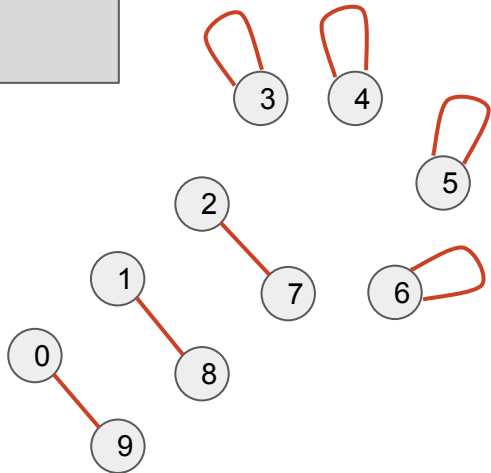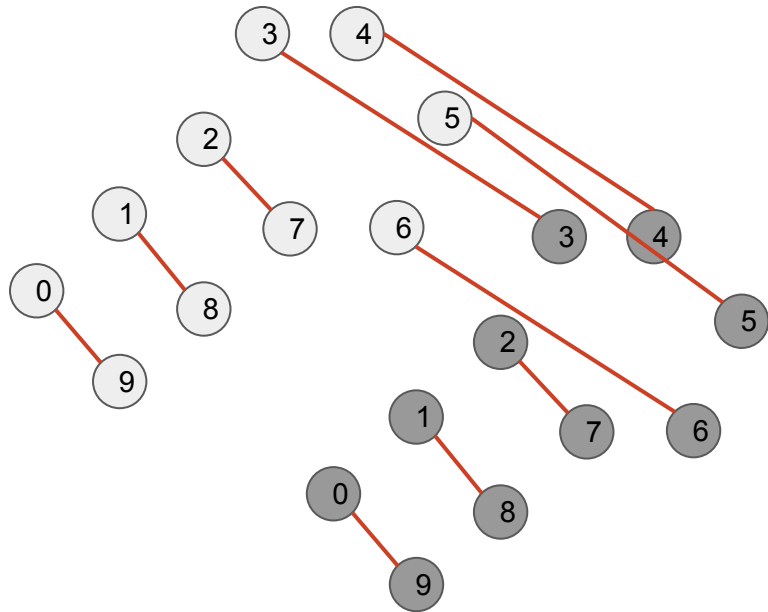

Supplement: Supplementary file 1 — Additional file 1. An example for how we model the problem for Blossom.Assume the simple structure in the gray box (top left). The graphrepresentation for the pairings is shown in the left graph as eachpairing is an edge between two vertices. As the Blossom cannothandle self-loops (which are the unpaired bases), we create a copyof the graph (shown in the right graph with gray nodes) and converteach self-loop into an edge between the node and its copy. For thesake of simplicity, we set k = 1 in this example. [file 12859_2021_4540_MOESM1_ESM.pdf]

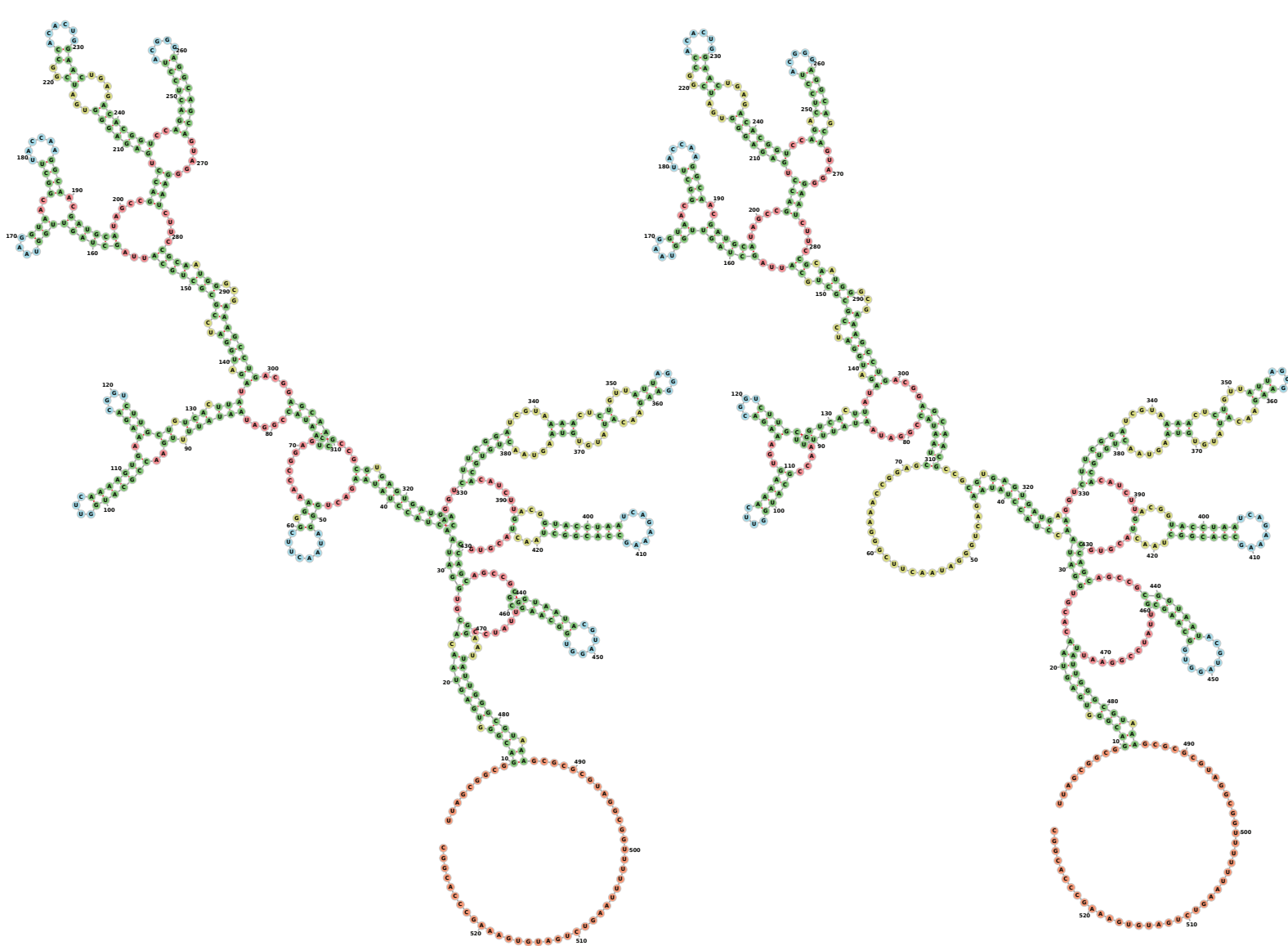

(a) Target

(b) CNNFold-mix

Supplement: Supplementary file 2 — Additional file 2. DQ923214 from 16sRNA family, accuracy of CNNFold-mix is 97.1\% F1-score.(a) is the target structure and (b) is our prediction. [file 12859_2021_4540_MOESM2_ESM.pdf]

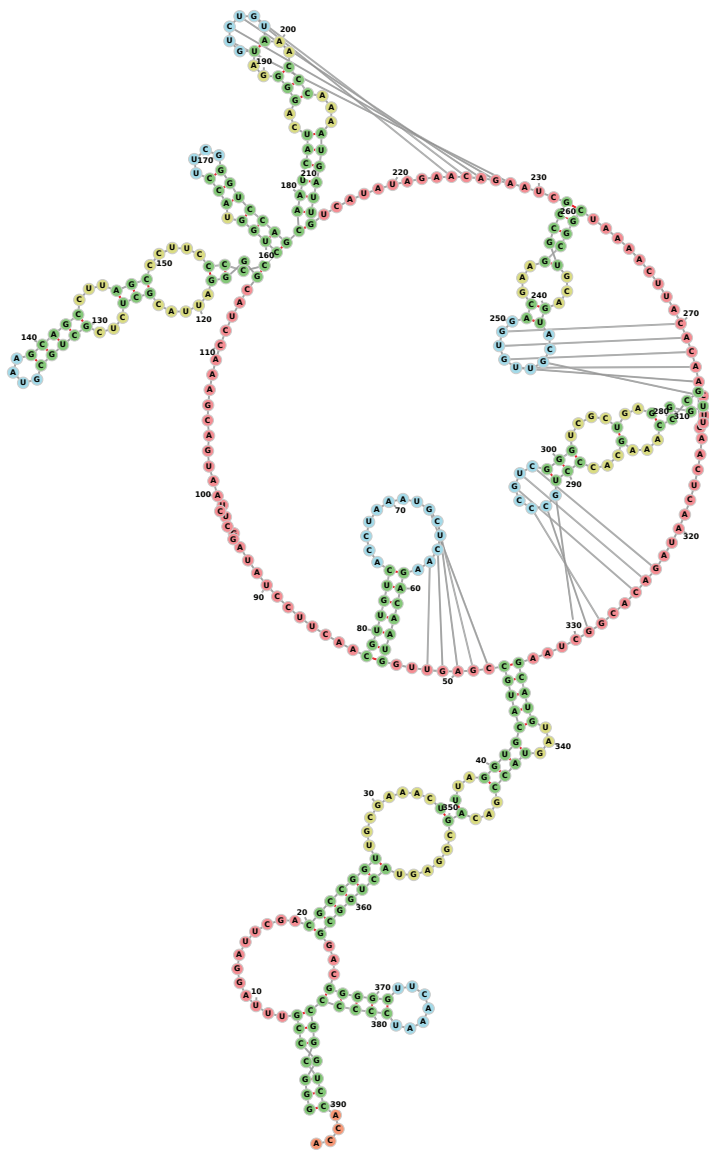

(a) Target

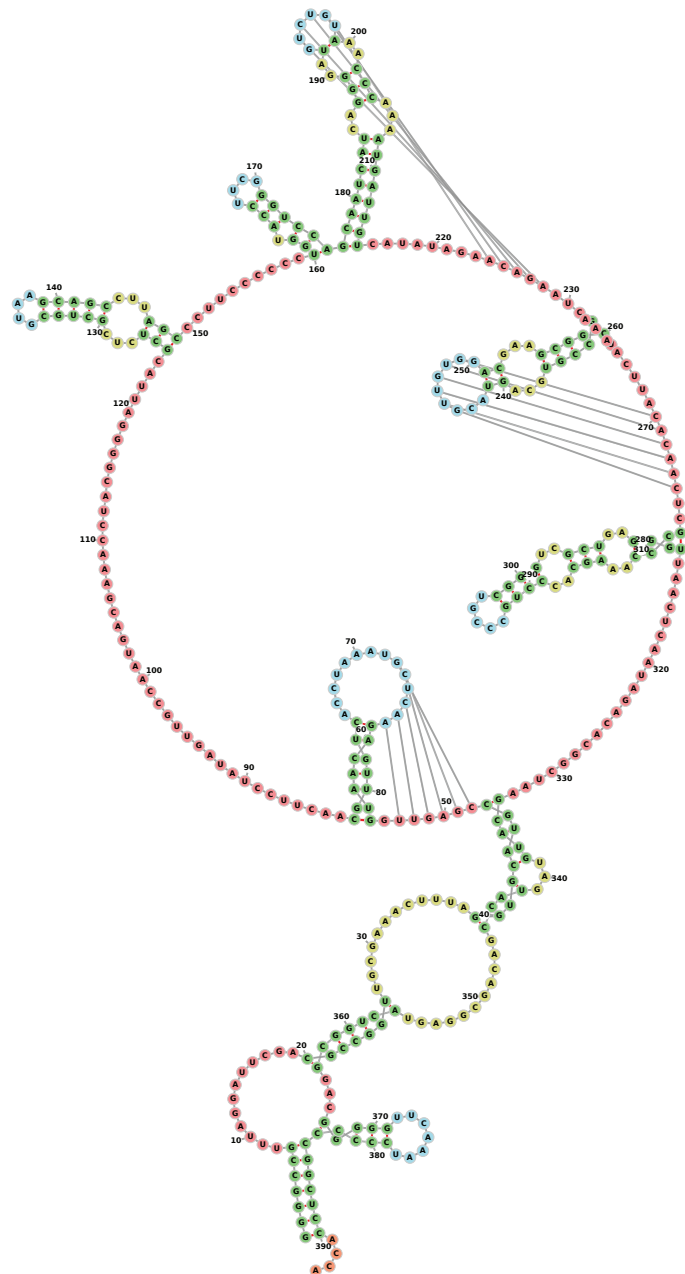

(b) CNNFold-mix

Supplement: Supplementary file 3 — Additional file 3. CP000076 with pseudoknots, F1-score of CNNFold-mix is 90.8\% F1-score.(a) is the target structure and (b) is our prediction. [file 12859_2021_4540_MOESM3_ESM.pdf]

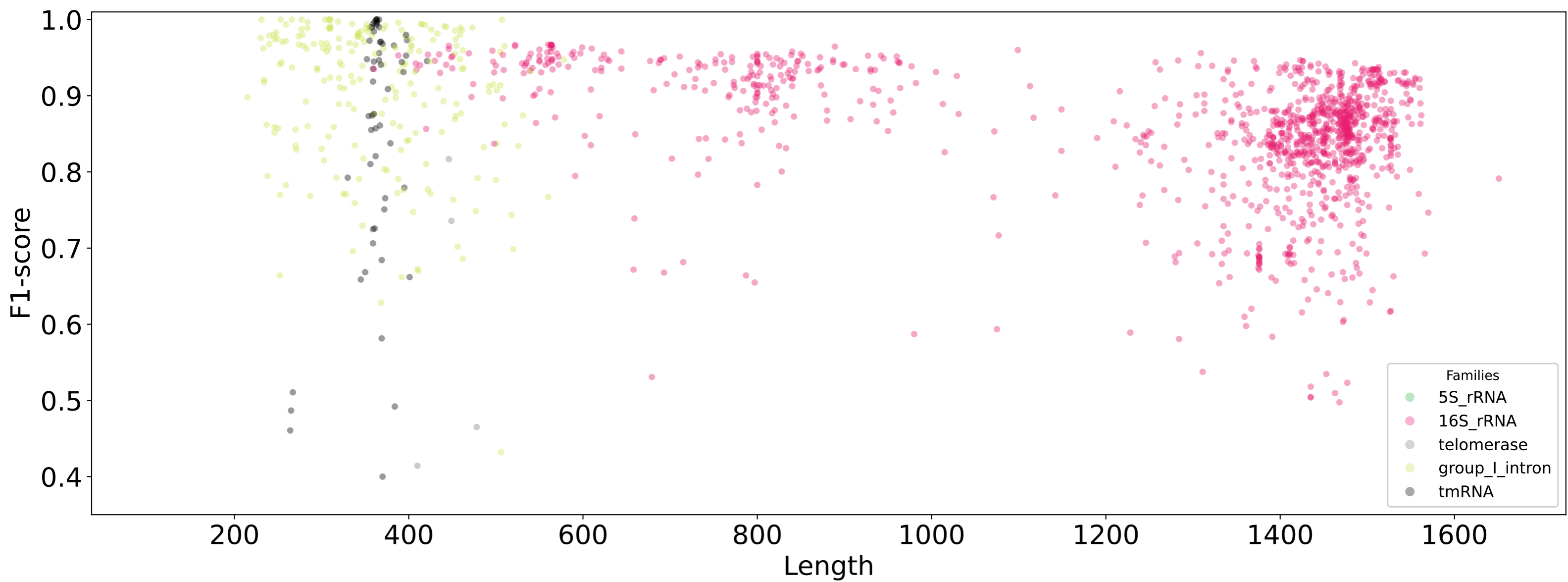

Supplement: Supplementary file 4 — Additional file 4. Scatter plot of the per-sequence F1-scores against the sequence lengths for pseudoknotted structures. Each point represents a sample and the model is CNNFold-mix. Coloursindicate sequences from 6 RNA families from RSA-ts. [file 12859_2021_4540_MOESM4_ESM.pdf]
